# Mental Derangement and Outward Surroundings

**Published:** 1890-10-04

**Authors:** P. H. Good

**Affiliations:** Portsmouth


### EDITOR'S LETTER-BOX.

[Our Correspondents are reminded that prolixity is a great bar to publication, and that brevity of style and conciseness of statement greatly facilitate early insertion.]

### MENTAL DERANGEMENT AND OUTWARD SURROUNDINGS.

SIR,—The question of how far our outward surroundings may colour and affect not our mental perceptions themselves, but the exercise of them, seems to me in this rapid age—rapid, I mean, in development of ideas—worthy of consideration. How often may the initial stages of mental disease be traced to the unnoticed but continued action of the patients' outward surroundings! Take, for instance, the case of an ordinary young lady we will say, from 16 to 20 years of age, who has lived all her life in a high church atmosphere. At this period, the most gushing period of her life, she begins to exhibit some unexpected peculiarities. She secludes herself from her lay friends to pass her time in what she believes to be prayer and meditation, becoming at the same time very fanciful about the state of her feelings, and reserved and snappish to her best friends and to those who love her most. At the same time she has selected some clergyman to whom she is willing enough to pour out her whole soul, and in whose ear she is always lamenting that she is not living up to his ideal and standard, to which she thinks he has attained. Meanwhile she has lost all interest and pleasure in the pursuits and enjoyments of most young ladies of her age and station in life. Her friends, although greatly distressed, see nothing to demand medical attention, but summon to their aid very likely some clergyman, the very last man (under the circumstances) they ought to call in. Every physician, I suppose, in active practice in fashionable localities can recollect such cases that have passed into evident insanity, and whose friends have expressed the utmost surprise that she should have "got into such a state." Ignorant of the meaning of the early symptoms when they began to manifest themselves, and little dreaming of the fact of her mind, little by little, giving way under the daily friction of her "æsthetic" religion—or, rather, what passed for religion—they have awoke to the reality of her condition when it is all too late. This may be cited as an instance of how, even in the upper and easier classes, the initial stages of mental derangement may be traced to the continual wear of the spirit by outward surroundings. What, then, must the effect be of the grimy surroundings of the less fortunate classes! Surely the conditions of life in this nineteenth century of ours, with its keen competition for bare existence or for exhausting sensational excitements, present problems for the philanthropist, the physician, and the divine. At this present stage, when our knowledge is so dim, so limited, and obscure, our duty is to wait and to watch the broadening streak of purer light, and while making a note of idiosyncracies, also to make one of surroundings, even those surroundings that from their apparently advantageous and outwardly beneficial belongings would seem to combat previous theory.

P. H. GOOD, M.A.

Portsmouth.

